# Genome-wide variation in the pinewood nematode *Bursaphelenchus xylophilus* and its relationship with pathogenic traits

**DOI:** 10.1186/s12864-015-2085-0

**Published:** 2015-10-23

**Authors:** Juan E. Palomares-Rius, Isheng J. Tsai, Nurul Karim, Mitsuteru Akiba, Tetsuro Kato, Haruhiko Maruyama, Yuko Takeuchi, Taisei Kikuchi

**Affiliations:** Division of Parasitology, Faculty of Medicine, University of Miyazaki, Miyazaki, 889-1692 Japan; Biodiversity Research Center, Academia Sinica, Taipei, 11529 Taiwan; Department of Biochemistry and Molecular Biology, Jahangirnagar University, Savar, Dhaka, 1342 Bangladesh; Forestry and Forest Products Research Institute, Tsukuba, 305-8689 Japan; Laboratory of Terrestrial Microbial Ecology, Graduate School of Agriculture, Kyoto University, Kyoto, 606-8502 Japan

**Keywords:** *B. xylophilus*, Pine wilt disease, Genome variation, Pathogenesis, Isolate, Inbred line

## Abstract

**Background:**

*Bursaphelenchus xylophilus* is an emerging pathogenic nematode that is responsible for a devastating epidemic of pine wilt disease across Asia and Europe. In this study, we report the first genome-wide variation analysis of the nematode with an aim to obtain a full picture of its diversity.

**Methods:**

We sequenced six key *B. xylophilus* strains using Illumina HiSeq sequencer. All the strains were isolated in Japan and have been widely used in previous studies. Detection of genomic variations were done by mapping the reads to the reference genome.

**Results:**

Over 3 Mb of genetic variations, accounting for 4.1 % of the total genome, were detected as single nucleotide polymorphisms or small indels, suggesting multiple introductions of this invaded species from its native area into the country. The high level of genetic diversity of the pine wood nematode was related to its pathogenicity and ecological trait differences. Moreover, we identified a gene set affected by genomic variation, and functional annotation of those genes indicated that some of them had potential roles in pathogenesis.

**Conclusions:**

This study provides an important resource for understanding the population structure, pathogenicity and evolutionary ecology of the nematode, and further analysis based on this study with geographically diverse *B. xylophilus* populations will greatly accelerate our understanding of the complex evolutionary/epidemic history of this emerging pathogen.

**Electronic supplementary material:**

The online version of this article (doi:10.1186/s12864-015-2085-0) contains supplementary material, which is available to authorized users.

## Background

Pine wilt disease (PWD) is one of the most serious global conifer diseases affecting native species of *Pinus* from the Far East forestlands to some parts of Europe [1]. The pine wood nematode (PWN) *Bursaphelenchus xylophilus* is the causal agent of PWD. PWN kills infected trees usually within a year of infection. This nematode is believed to be native to North America and causes little damage to the *Pinus* species in those regions. However, once introduced into a susceptible tree area, prevention of the spread of *B. xylophilus* becomes increasingly difficult because the nematode is vectored by the adult (flying) stage of *Monochamus* beetles [2].

*Bursaphelenchus xylophilus* is thought to have been introduced into Japan approximately 100 years ago from North America [2]. The first description of PWD in the Nagasaki Prefecture in Japan occurred in 1905 [3], although *B. xylophilus* was not identified as the causal agent of the disease until 1971 [4]. PWD has since spread to other East Asian countries, such as China Taiwan, and Korea in 1982, 1987 and 1988, respectively [5–7]. Moreover, PWD was found in Portugal in 1999 [8] and has now spread to Spain [9].

Over the past two decades, several molecular techniques have been applied to assess the genetic variability of *B. xylophilus* with the aim of revealing the origins and patterns of spread of this pathogenic nematode [10–17]. Some of these reports suggested that isolates from non-native areas (Japan, China, Korea and Portugal) exhibited less genetic diversity than those from the native area (North America) [13, 18]. However, a significant degree of genetic diversity or even greater genetic diversity than native isolates was also observed in non-native isolates [10, 15]. Therefore, it remains inconclusive regarding the genetic diversity of PWN from those studies, which demands a genome-wide analysis to have a full picture of this nematode diversity.

The genome of *B. xylophilus* was sequenced in 2011 [19] and permit the investigation of population structures by genome-wide analyses and the identification of genetic factors involved in its key biological processes and parasitism. The genetic diversity of *B. xylophilus* isolates from different geographic locations was recently assessed using single nucleotide polymorphisms (SNPs) from transcriptome data [20]. However, the genome-wide variations between the populations of *B. xylophilus* remains unknown, which presumed to have different geographical origins and possess different phenotypic characteristics such as degree of pathogenicity (virulence) and reproductive ability on fungi. Identification of such variations will shed light on the molecular mechanism of *B. xylophilus* pathogenicity, as well as clarify the complex evolutionary and epidemic history of this nematode.

During the history of PWD in Japan, a number of *B. xylophilus* strains have been isolated from the natural environment. Extensive studies were performed on these strains of *B. xylophilus* to assess their pathogenic characteristics and ecological traits [21–24]. Increasing phenotypic information concerning the isolates of *B. xylophilus* in Japan encouraged us to explore the intra-species genomic variation of this nematode.

In this study, we selected and sequenced six representative strains isolated in Japan that were used widely in previous studies (Table 1). We have shown that between strains of *B. xylophilus* genomes exhibit high genetic diversity, indicating multiple origins of *B. xylophilus* populations in Japan. To the best of our knowledge, this study represents the first genome-wide attempt to estimate the variation in *B. xylophilus* populations.

**Table 1:** Origin and ecological traits of *B. xylophilus* strains used in this study

Strains	Origin	Line/ Isolate	Virulence^1^	Reproductive ability on fungus^2^	Reproductive ability on *Pinus* trees^3^
C14-5	Chiba, Japan	Isolate	+^5,6^	+^5,6,7^	+^5,6^
OKD1-F7	OKD-1 isolate (Okayama, Japan)	Inbred line	+^4^	+^4^	+^4^
S10-P3	S10 isolate (Shimane, Japan)	Inbred line	++^4^	++++^4^	++^4^
S10-P9	S10 isolate (Shimane, Japan)	Inbred line	++++^4^	++++^4^	++++^4^
T4	Iwate, Japan	Isolate	++++^6^	++++^6,7^	-
Ka4C1	Ka-4 isolate (Ibaraki, Japan)	Inbred line	++++^5^	++++^5^	++++^5^

^1^Virulence is defined as degree of pathogenicity. Mortality of Pinus thunbergii seedlings: + < 25 %; ++: 25-50 % and ++++: > 75-100 %. ^2^Percentage increase of initial population cultured on *Botrytis cinerea* after 5-8 days; +: < 1.1x; ++++: > 20x initial population. ^3^Percentage increase of initial population in inoculated *P. thunbergii* seedlings after 28 days; +: < 1.1x; ++: 1.1-6x; ++++: > 20x initial population; - : no information available. Information was obtained from ^4^Shinya et al., 2012; ^5^Aikawa and Kikuchi, 2007, information for Ka4 original isolate; ^6^Mota et al., 2006; ^7^Wang et al., 2005

## Results

### Inter-strain genomic variation in Bursaphelenchus xylophilus

In order to investigate the genomic diversity of *B. xylophilus*, we re-sequenced six representative strains with different phenotypic or ecological traits (Table 1). The genome of *B. xylophilus* was revealed to be highly variable between the strains studied. We detected 3,040,397 variant positions, which accounted for 4.1 % of the total genome (Table 2, Additional file 1: Table S1). Most of the variants were SNPs (2,772,939 positions), and a smaller number of positions had small indels, comprising 160,655 and 164,133 positions with insertions or deletions of 2–16 bp, respectively. Of these indels, 34,115 could be possible errors, as they were located adjacent to homopolymer regions longer than 5 bp.

**Table 2:** Statistics of the variants found in *B. xylophilus* strains

		Strains				
	ALL	C14-5	OKD1-F7	S10-P3	S10-P9	T4
Genomic positions with variants	3,040,397	1,933,181	1,859,643	945,217	944,920	885,905
Genomic positions with SNPs	2,772,939	1,754,649	1,683,010	846,799	846,616	795,391
Homozygotic SNP ratio (%)	95.28	93.81	95.90	95.96	95.98	95.74
SNPs (transitions)		1,037,660	991,210	496,779	496,806	467,365
SNPs (transversions)		719,934	694,556	351,645	351,459	329,351
Ratio transitions/transversions		1.44	1.43	1.41	1.41	1.42

All variant numbers were from comparisons to the reference genome

The SNPs and indels identified were validated by polymerase chain reaction (PCR), followed by Sanger sequencing. A selected set of 34 variants (20 SNPs and 14 small indels) was used for the experimental validation. All SNPs and small indels were validated by the experiment, excluding one position with a homo/hetero difference, suggesting that our variance predictions were highly accurate (Additional file 1: Table S2).

**Fig. 1:**
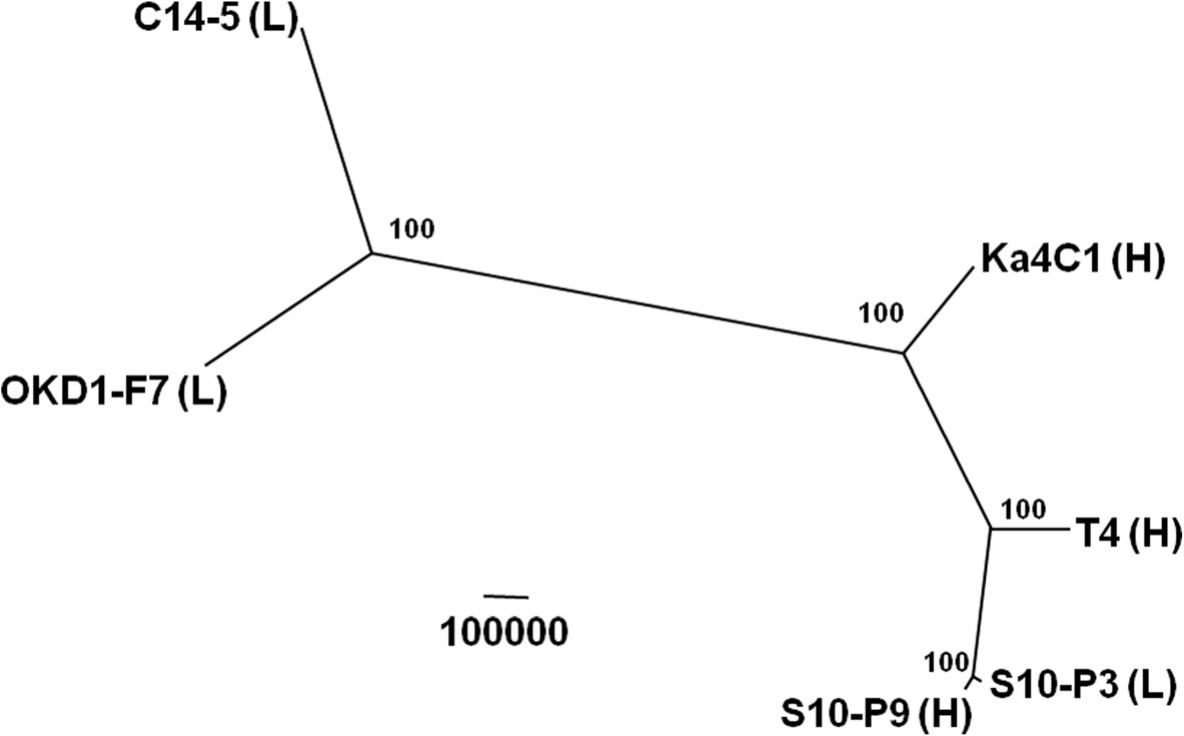
A maximum parsimony tree from single nucleotide polymorphisms (SNPs) using PAUP*4b10 with 100 bootstrap resampling replicates (*shown at the branches*). The scale bar represents the number of homologous substitutions. (H) and (L) after strain names indicate high virulence and low virulence, respectively

A maximum parsimony tree was generated based on 2,772,939 genomic positions with SNP variants. The tree displayed good support for all samples studied (Fig. 1). The samples were divided into two major groups, one comprising C14-5 and OKD1-F7 and the other comprising the remaining four samples including the reference strain Ka4C1. An extremely close relationship was observed between S10-P3 and S10-P9, consistent with the fact that these were inbred lines derived from the same parents, whereas T4 was positioned distinctly from them. More than 1.8 million variants were detected in C14-5 and OKD1-F7 compared with the reference genome; whereas, smaller, but still high, numbers of variants (more than 0.8 million) were observed for the other three strains (Table 2). Most of the variants (>95 %) identified were homozygotes (fixed) in each strain (Table 2). In the three strains that had undergone inbreeding (OKD1-F7, S10-P3 and S10-P9), 95.90, 95.96 and 95.98 % of the variants were homozygous, respectively. In the two isolates that did not experience inbreeding (C14-5 and T4), 93.81 and 95.74 %, respectively, of the variants were homozygous.

**Fig. 2:**
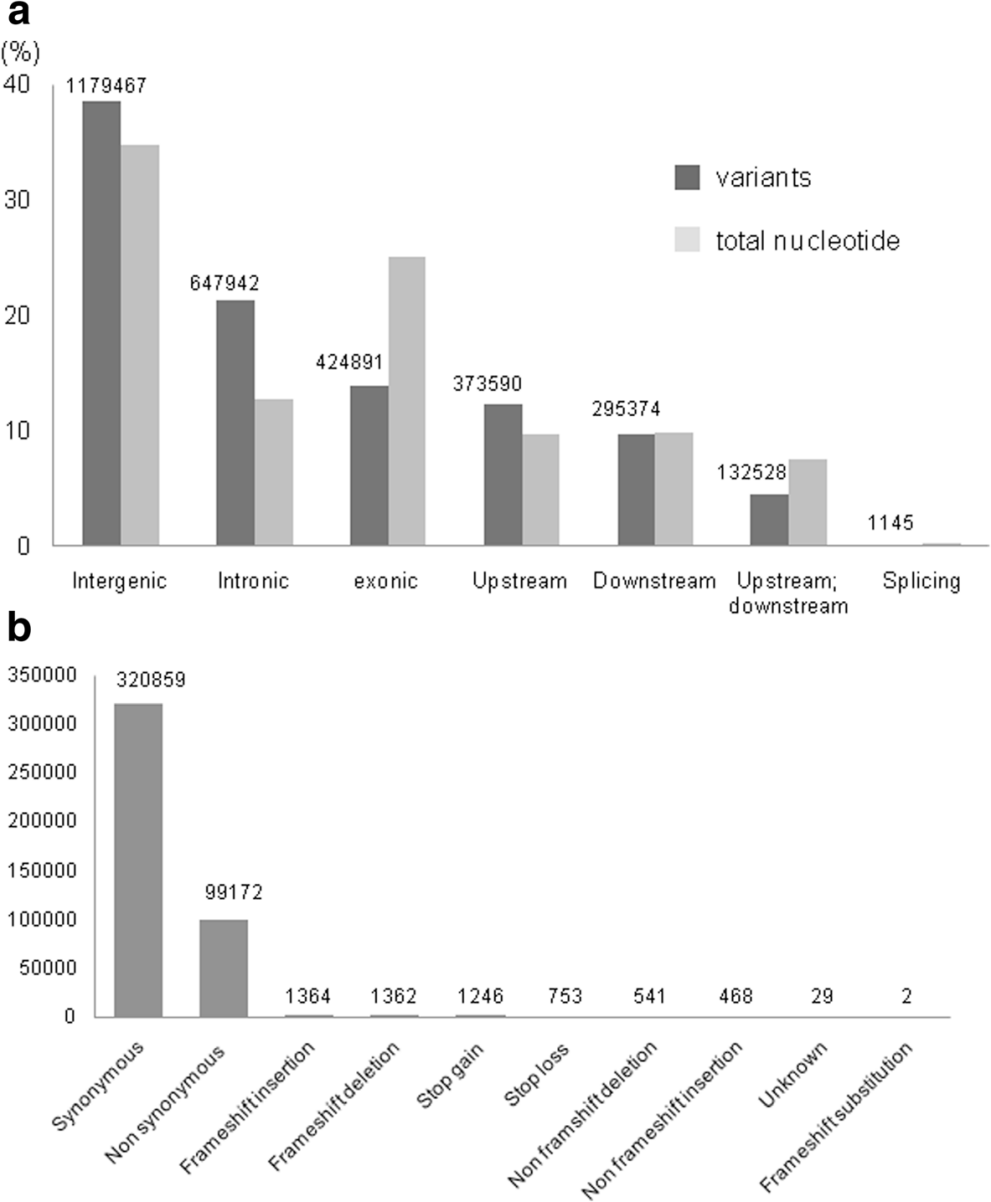
**a** Variant position percentages across sequence classes. The ANNOVAR program was used to classify variant positions. Intergenic: variant is in the intergenic region, not included in Upstream or Downstream, Intronic: variant overlaps an intron, Exonic: variant overlaps a coding region, Upstream: variant overlaps 1-Kb region upstream of the transcription start site, Downstream: variant overlaps 1-Kb region downstream of the transcription end site, Splicing: variant is within 2 bp of a splicing junction. The absolute numbers of variants were shown above the bars. Genome percentages of the same classes are shown alongside. **b** Effects of the Exonic variants. Synonymous : a single nucleotide change that does not cause an amino acid change, Non synonymous : a single nucleotide change that causes an amino acid change, Frameshift insertion/deletion: an insertion or deletion of one or more nucleotides that cause frameshift changes to proteins, Stop gain/loss: a nonsynonymous SNP or indel that leads to the immediate creation/elimination of a stop codon at the variant site, Frameshift substitution: a block substitution (not insertion or deletion) of one or more nucleotides that cause frameshift changes, unknown: unknown function (caused by various errors in the gene structure definition in the database file). Each position can have more than one alternative variant and the effect could be different

Comparisons of variant positions with the reference gene models revealed that 38.6 and 21.2 % of the variations were located in intergenic and intronic regions, respectively, representing higher frequencies than the ratios of the individual nucleotides in the total genome (Fig. 2a). Exonic, upstream/downstream and splicing regions had fewer variants compared with the ratios of the individual nucleotides in the total genome (Fig. 2a). Among the exonic variants, there were 320,859 synonymous and 99,172 non-synonymous SNPs. There were fewer indels that could introduce frameshifts (1,364 insertions, 1,362 deletions and 2 substitutions) (Fig. 2b). There were 1,246 stop gain and 753 stop loss variants (Fig. 2b). Distribution patterns of variants and possible effects were similar between the strains (Additional file 2: Figure. S1 and Additional file 2: Figure. S2).

**Fig. 3:**
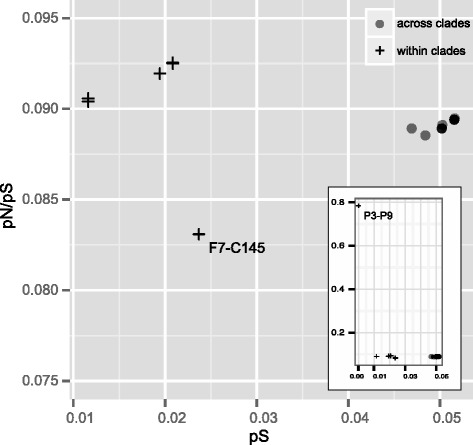
Relationship of the pN/pS ratio and synonymous site diversity (pS) of all pair-wise comparisons of six *B. xylophilus* strains

Investigation of the number of synonymous and non-synonymous substitutions provides information about the degree of selection during the evolution of a particular population. To determine the genetic diversity in coding regions, we calculated the number of synonymous substitutions per synonymous site (pS) as well as the number of non-synonymous substitutions per non-synonymous site (pN). We found a mean synonymous site diversity μ_si_ = 3.39 × 10^−2^ and a mean non-synonymous site diversity μ_ns_ = 3.03 × 10^−3^. The resulting value of pN/pS of 0.0893 suggested that strong purifying selection acting on the coding regions of these nematodes. The pair-wise comparisons illustrated that pN/pS ratios varied from 0.0831 to 0.0925, with an exceptionally high value (0.785) obtained from the comparison between S10-P3 and S10-P9 (Fig. 3). As observed in *Pristionchus pacificus* [25], a lower pN/pS ratio was observed in across-clade comparisons (i.e. larger distance) than in within-clade comparisons. However, the pN/pS ratio of the intra-clade comparison between C14-5 and OKD1-F7 deviated strongly from this observation.

### Frameshift and stop codon variants

Genomic variants that introduce frameshift or stop codon mutations can have serious effects on protein structures and functions. The results of a functional enrichment test for frameshift and stop codon variants are shown in Additional file 1: Table S3 and Additional file 1: Table S4, respectively. For the frameshift variants, six and five Gene Ontology (GO) terms were significantly enriched in C14-5 and OKD1-F7, respectively, whereas only two GO terms were enriched in the other three strains (Additional file 1: Table S3). Several GO terms were related to proteolysis and peptidase inhibitor activity. Metallopeptidase activity was over-represented in three strains (OKD1-F7, C14-5 and T4), aspartic-type endopeptidase activity was over-represented in two strains (OKD1-F7 and C14-5) and cysteine-type endopeptidases inhibitor activity was over-represented in four strains (OKD1-F7, C14-5, S10-P3 and S10-P9). Hydrolase activity acting on carbon-nitrogen bonds (but not peptide bonds) and cadmium ion transmembrane transporter activity were each over-represented in one strain (T4 or OKD1-F7, respectively). In addition, voltage-gated chloride channel activity was over-represented in three strains (C14-5, S10-P3 and S10-P9).

Similarly to the frameshift variants, for the stop codon variants, more over-represented pathways were found in OKD1-F7 (4) and C14-5 (4) than in S10-P3 (3), S10-P9 (3) and T4 (1). Metallopeptidase activity and alpha-trehalase activity were over-represented in OKD1-F7 and C14-5. Flavin mononucleotide binding was over-represented in T4, S10-P3 and S10-P9 (Additional file 1: Table S4). Acetylcholine-activated, cation-selective channel activity and dopamine beta monooxygenase activity were over-represented in S10-P3 and S10-P9 (Additional file 1: Table S4).

### De novo assemblies using un-mapped reads

Unmapped reads of each sample were assembled independently from the reference. Filtering by length (longer than 200 bp) and by overlapping ends (>50 bp) with the reference genome was applied to avoid any contaminating sequences, as described previously [26]. The total lengths of the assemblies were similar between the strains, varying from 260,975 (T4) to 342,305 bp (C14-5) (Additional file 1: Table S5). The numbers of contigs generated ranged 491–648, with the highest number identified for S10-P9 and the lowest found for T4 (Additional file 1: Table S5).

**Fig. 4:**
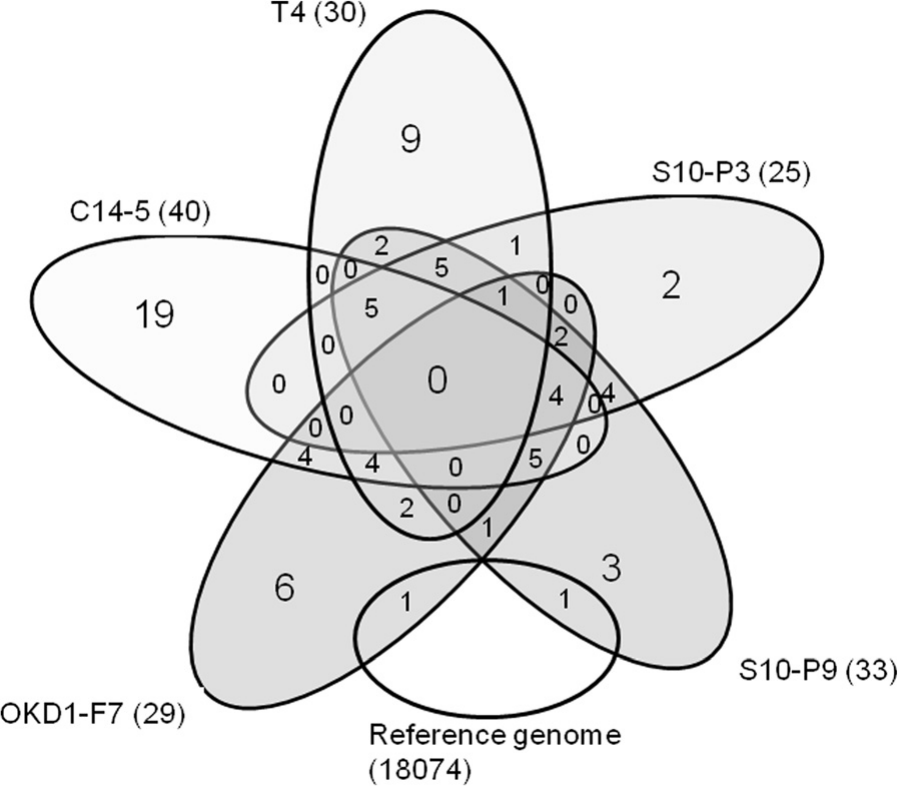
Venn diagram showing the number of unique and overlapping genes predicted in the un-mapped de-novo assemblies between the strains

Protein-coding genes were predicted on the assemblies using Augustus, with parameters optimised for *B. xylophilus* [19]. The numbers of predicted genes varied from 25 to 40 (Fig. 4 and Additional file 1: Table S5). Some genes were unique (as determined by using a ≤ 90 % similarity threshold) to each sample and the reference gene models, with the highest number and proportion in C14-5 (19 genes and 47.5 % of all of its *de novo* genes) and the lowest number and proportion in S10-P3 (2 genes and 8 % of all of its *de novo* genes) (Fig. 4). Several genes were found in more than one sample (Fig. 4). Annotations of those genes, based on Blast2Go software, are shown in Additional file 3: Table S6. Three proteins in S10-P3 were not found in S10-P9, and 11 proteins in S10-P9 were not found in S10-P3. Two and four of these proteins were unique in strains S10-P3 and S10-P9, respectively. Blast2GO analysis of the protein sequences that differed between S10-P3 and S10-P9 are shown in Table 3. Two proteins have signal peptides for secretion, whereas the other two have transmembrane domains. The unique genes in S10-P3 are related to the transferase activity of acyl groups (other than amino-acyl groups), helicase activity or binding activities. The unique genes in S10-P9 are related to N-acetyltransferase activity, cysteine-type peptidase activity and transport.

**Table 3:** Genes in the un-mapped read assemblies that were different between S10-P3 and S10-P9

Gene ID	Blast tophit (organism)	E-value	Coverage (%)	GO annotation	Signal-*P*
Genes present in S10-P3 and absent in S10-P9					
g53.t1_Ip1476*	db module family protein	2.45E-33	65.7	F: transferase activity; F: transferase activity, transferring acyl groups other than amino-acyl groups; F: transferase activity, transferring acyl groups	
g126.t1_Ip1476*	dna helicase	1.51E-06	50.4	F: helicase activity; C: chloroplast envelope; C: chloroplast; F: ATP binding; F: nucleotide binding; F: nucleoside-triphosphatase activity; F: nucleic acid binding; F:ATP-dependent 5’-3’ DNA helicase activity; C: plastid	
g80.t1_Ip1475	Integrase domain-containing protein	5.90E-17	59.45	F: binding	
Genes present in S10-P9 and absent in S10-P3					
g5.t1_Ip1474	No hit				
g129.t1_Ip1474	Protein isoform a	4.33E-10	42.7	F: N-acetyltransferase activity; P: embryo development ending in birth or egg hatching	
g131.t1_Ip1475	Cysteine proteinase rd19a-like	8.24E-07	55.45	P: proteolysis; F: hydrolase activity; F: cysteine-type peptidase activity; F: peptidase activity	SignalP-noTM (EUK)
g17.t1_Ip1477*	Syntaxin and target snare coiled-coil region domain-containing protein	2.30E-42	59.15	P: transport; P: cell-cell signaling; P: biological process	
g27.t1_Ip1477	wd repeat-containing protein 5	3.98E-174	83.2	P: multicellular organismal development; F: transferase activity; C: nucleoplasm; C: protein complex;; P: regulation of biological process; P: cellular protein modification process; P: organelle organization; F: protein binding	
g29.t1_Ip1477	Transthyretin domain-containing protein	2.46E-32	61.95	C: integral to membrane; C: extracellular space	SignalP-noTM (EUK)
g77.t1_Ip1477*	No hit	-	-	-	
g87.t1_Ip1477*	No hit	-	-	-	
g112.t1_Ip1477*	No hit	-	-	-	

*: genes unique to the specified sample, -: no information available

### Identification of copy number variations (CNVs)

CNVs represent a form of structural variation corresponding to relatively large regions of the genome that have been deleted (fewer than the normal number) or duplicated (more than the normal number) on certain chromosomes. In addition to identifying SNPs and small indels, we also investigated CNVs to determine whether there were large missing or duplicated parts in the *B. xylophilus* genomes. Compared with the reference genome, 4, 6.4, 2.9, 5.5 and 4.2 % of the genomes of C14-5, OKD1-F7, T4, S10-P3 and S10-P9, respectively, have large insertions or deletions ranging in size from 1–31 kb (Additional file 1: Table S7).

### Variants specific to low-virulence strains

Isolate C14-5 and OKD1 are relatively closely related [23], and they share similar characteristics with each other, including low virulence to the host and a slower life cycle (Table 1). Our phylogenetic analysis based on whole SNP positions confirmed that these strains were closely related to each other and distantly related to the other strains (Fig. 1). These two strains shared 1,285,536 common variants compared with the reference genome, which accounted for more than half of the total variants in each strain. Most of the shared variants (97.8 %) were homozygotic, and 153,812 (15.5 %) were exonic. Those exonic variants caused frameshift deletion/insertion (849 variants) and stop codon mutations (641 variants). Homozygotic variations affected 532 genes as frameshift variants, 507 genes as stop codon variants and 8,870 genes as non-synonymous variants. Metalloendopeptidase activity and peptide-transporting ATPase activity were also enriched among these frameshift changes (Additional file 1: Table S8), and the structural constituent of muscle and endopeptidase activity were over-represented in the stop codon changes (Additional file 1: Table S8).

### Comparison between two inbred lines from the same parents

**Fig. 5:**
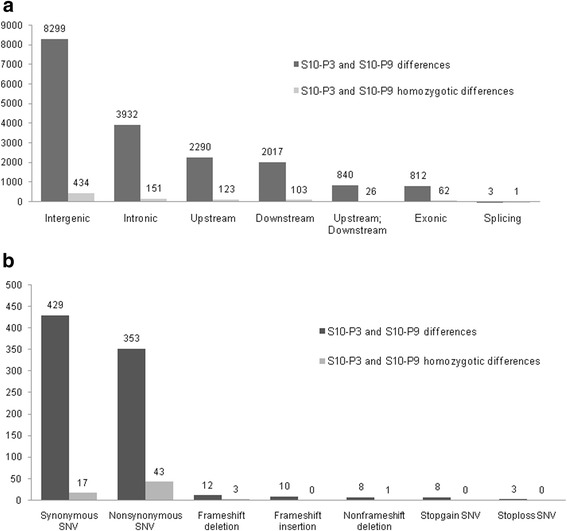
**a** Variant position and effect in differential variants between S10-P3 and S10-P3. Variant position in at least one of the samples studied using the ANNOVAR program. **b** Variant effects of the exonic variants between S10-P3 and S10-P3. Please refer to the legend for Fig. 2 for the descriptions

Of particular interest, strains S10-P3 and S10-P9 were compared to identify genes related to differences in pathogenicity (virulence) because these inbred lines originated from the same parent pair [24]. This comparison revealed that only 18,413 genomic positions were different between the two inbred lines, which accounted for 0.02 % of the genome. Among them, only 904 variants (4.91 %) were homozygotic (Fig. 5). The number of exonic variants between the two lines was only 874, consisting primarily of synonymous and non-synonymous SNPs, accounting for 429 and 353 variants, respectively. There were 22 frameshift variants (12 insertions and 10 deletions), in which only three positions were homozygotic (Fig. 5). Functional annotation of these genes suggested their involvement in transport, protein phosphatase activity and phosphorylation activity (Table 4). All stop codon variations were heterozygous between the two lines. Homozygotic non-synonymous SNPs that differed between S10-P3 and S10-P9 were predicted to affect the functions of five genes (BUX.s00119.53, BUX.s0042.86, BUX.s00713.1105, BUX.s00770.54 and BUX.s01281.204) based on analysis using the SIFT algorithm [27] and Align GVGD [28]. In particular, BUX.s00770.54 and BUX.s00713.1105 have higher probabilities of effect (class C25 for Align GVGD program).

**Table 4:** Genes affected differently between S10-P3 and S10-P9 by frameshift changes, stop codon changes and non-synonymous SNPs

Gene ID	S10-P3^a^	S10-P9^a^	BLAST tophit	E-value	Coverage (%)	GO Annotation	Signal-*P*
Frameshift changes							
BUX.s00036.207	0/1	1/1	Filamin abp280 repeat-containing domain protein	1.06E-60	48.27	F: transferase activity, transferring phosphorus-containing groups; F: kinase activity; P: phosphorylation	
BUX.s00116.453^*b*^	1/0	0/0	Na (+)–dependent inorganic phosphate co-transporter	6.31E-101	55.9	C: cell; P: transport	SignalP-NN (euk)
BUX.s00172.1	0/1	0/0	protein srt-52	3.75E-16	55.9	P: biological_process	
BUX.s00358.10	0/1	1/1	Degenerin unc-8	2.28E-18	51.75	C: cell; C: plasma membrane; P: biological_process; F: ion channel activity; P: behavior; P: response to external stimulus; P: response to abiotic stimulus; P: ion transport	
BUX.s00460.315	0/1	1/1	Protein ugt-15	4.018E-71	50.5	P: metabolic process	SignalP-NN (euk)
BUX.s01066.84	0/1	1/1	Protein kinase	1.90E-05	51.82	F: ATP binding; F: protein kinase activity; F: transferase activity; P: protein phosphorylation; F: transferase activity, transferring phosphorus-containing groups; F: protein serine/threonine kinase activity; F: kinase activity; P: phosphorylation	
BUX.s01092.23	0/1	0/0	Protein nkcc-isoform e	9.34E-28	72.8	C: cell; P: transport	
BUX.s01133.2	1/1	0/0	Protein-tyrosine phosphatase-containing protein	3.76E-49	56.3	F: phosphoprotein phosphatase activity; P: cellular protein modification process	
BUX.s01144.298	0/0	1/1	Discoidin domain-containing receptor 2-like	2.87E-113	52.25	P: cell adhesion; F: ATP binding; P: protein phosphorylation; F: protein tyrosine kinase activity	
BUX.s01144.7	0/1	0/0	Protein lgx- isoform a	1.18E-119	51.5	F: protein binding; P: carbohydrate metabolic process; F: catalytic activity; F: metal ion binding	
BUX.s01147.248	1/1	0/1	Cysteine-rich motor neuron 1 protein	6.71E-19	46.65	P: multicellular organismal development; F: protein binding	
Stop changes							
BUX.s00116.766	1/1	0/1	Hypothetical protein CAEBREN_14873 (protein isoform a)	2.54E-31	48.95	C: membrane; P: molting cycle, collagen and cuticulin-based cuticle; P: locomotion; P: positive regulation of growth rate; P: nematode larval development; P: growth	
BUX.s00351.27	0/0	0/1	Uncharacterised oxidoreductase–like	7.92E-51	57.7	P: metabolic process; F: catalytic activity	
BUX.s00460.265	1/1	0/1	gdp-d-glucose phosphorylase 1	9.86E-127	58.35	C: cytoplasm; P: embryo development; F: transferase activity; P: carbohydrate metabolic process	
Genes with possible functional effect by homozygotic non-synonymous SNPs^c^							
BUX.s00119.53 (SIFT + align-GVGD)	1/1	0/0	Pot family protein	0.0E0	57.3	F: transporter activity; P: ion transport; F: ion channel activity; P: cell cycle; P: transport	
BUX.s00422.86 (SIFT + align-GVGD)	1/1	0/0	Kap family p-loop domain-containing protein	0.0E0	62.35	-	
BUX.s00713.1105 (SIFT + align-GVGD)	1/1	0/0	No hit	-	-	-	SignalP-NN (euk)
BUX.s00770.54 (SIFT + SIFT + align-GVGD, inter-medium (class C25))	1/1	0/0	Innexin unc-7	0.0E0	82.6	P: behavior; P: reproduction; P: regulation of biological process; C: cell; C: plasma membrane; P: biological_process; P: cellular component organization; F: transporter activity; P: ion transport; P: protein metabolic process; P: catabolic process	
BUX.s01281.204 (SIFT + SIFT + align-GVGD, inter-medium (class C25))	1/1	0/0	No hit	-	-	-	SignalP-NN (euk)

^a^0/0: equal to the genome reference, 0/1: heterozygous, 1/1: homozygotically different to the genome reference; ^b^variants found to be heterozygotic in the validation process; ^c^SIFT (first) and align-GVGD were used to find the putative effects on the proteins; Genes underlined are homozygotic at the SNP sites; −: no information available)

### Variants in the reference strain

The variant positions with homozygous alleles that were common in all samples sequenced, but different from the reference genome, were considered possible errors in the reference assembly or mutations that had occurred during the time gap between the two DNA preparations. Re-sequencing of the reference genome allowed us to identify 2171 genomic positions (574 SNPs and 1597 indels) that were different from the published genome [19]. The majority of them were homozygotic, and only two genomic positions had heterozygotic differences.

## Discussion

This is the first genome-wide attempt to understand the intra-species diversity of *B. xylophilus*. In this study, we sought to assess the extent of divergence among *B. xylophilus* genomes in Japan, in which the nematode was introduced from its native area. Furthermore, we attempted to relate the diversity to variability in phenotypic traits, such as virulence, reproductive ability on fungus and some ecological traits. We selected six representative strains of *B. xylophilus* isolated from different parts of Japan that have different phenotypic or ecological traits and that have been used in several previous studies (Table 1).

Our genome-wide analysis revealed that *B. xylophilus* had a significantly high degree of genome variation between the strains (4.1 % of the genome positions were variable as SNPs or indels). High levels of genome variations have also been observed in other nematodes, including *C. brenneri*, which exhibited 14.1 % of polymorphic synonymous sites between individuals [29], and two other *Caenorhabditis* nematodes (*C. remanei* and *Caenorhabditis* sp. 5) were determined to have hyper-diverse genomes (>5 %) [30]. However, the fact that all *B. xylophilus* strains used in this study were isolated in Japan and the observation of significant degrees of genomic variations between these strains highlighted the possibility that multiple introductions of this nematode occurred in Japan from the native area.

All nematode strains exhibited a high degree of homozygosity, even in samples that did not undergo inbreeding (T4 and C14-5) (Table 2). This can be explained by the founder effect, in which the loss of genetic variation occurs when a new population is established by an extremely small number of individuals, and the Wahlund effect, in which a reduction in heterozygosity occurs in a population caused by a subpopulation structure [14]. PWNs multiply explosively from a small number of infected nematodes within individual trees, and they have subpopulation structures in the pathogenic lifecycle. Moreover, the effect of strain maintenance in the laboratory, involving extremely short generation times and the transfer of a small number of nematodes to a new bottle, can decrease the genetic diversity within a population [17].

The pN/pS ratio in the *B. xylophilus* strains (0.0893 among all the strains) was extremely low compared with the ratio (0.32) found in *P. pacificus* [25]. This can be explained by the fact that *B. xylophilus* is a parasitic nematode and is likely to have experienced higher selective pressures in the host tree. Alternatively, this nematode has a large population size, as it can multiply to millions in the host tree once the host has died. This large population size can also render very effective purifying selection [31]. The much higher pN/pS ratio found in the S10-P3 and S10-P9 pair-wise comparison (0.785) is consistent with this explanation because the two inbred lines have not experienced that type of selection pressure after being separated in the inbred line generation process.

GO enrichment analysis revealed that the strains studied contain high levels of possible loss of function due to frameshift or stop codon variations in proteins related to proteolysis, which includes metallopeptidase activity, aspartic-type endopeptidase activity and cysteine-type endopeptidase inhibitor activity (Additional file 1: Table S3 and Additional file 1: Table S4). This is probably because the number of peptidase gene families in the *B. xylophilus* genome has expanded, comprising 581 genes, representing the biggest gene number of genes among sequenced nematodes [19]. The fact that such variants were enriched in these genes may suggest that regions with such expansions are also subject to change in terms of point mutations or small insertions and deletions.

Strains C14-5 and OKD1-F7 have distinct phenotypes and ecological traits from other *B. xylophilus* strains (Table 1). They multiply slowly on fungi and exhibit extremely low virulence to pine trees. Therefore, to survive in nature, they should have a strategy and lifecycle that are distinct from those of the typical pathogenic strains, and a hypothesis that their origin is different from other strains has been proposed. Isolating this type of population from the wild is difficult, and only two natural isolates have been reported to date; they are neither highly populous around Japan nor involved in the massive pine death in this country (Table 1). The phylogeny based on all SNP locations in the genome suggested that the two strains are only distantly related to the other strains, but are not closely related to each other within the clade (Fig. 1). These results suggested that similarly to the typical PWN populations, this type of population was also introduced several times into Japan, or they had been present for a long time in Japan before the typical PWN was introduced. Furthermore, the lower pN/pS ratio found in the comparison of C14-5 and OKD1-F7 (0.0831) than in the other pair-wise comparisons (Fig. 3) also suggested that these strains may have a different lifestyle in nature, in which they experienced different types of selective pressures compared with the other high-virulence strains.

Our analysis revealed that two strains (C14-5 and OKD1-F7) shared many SNPs when compared with the other strains. GO enrichment analysis suggested several aspartic-type endopeptidases would display loss of function in C14-5 and OKD1-F7 (Additional file 1: Table S3). Other more specific activities, such as hydrolase activity acting on carbon-nitrogen bonds (but not peptides) in OKD1-F7 or cystathionine gamma-lyase activity in both C14-5 and OKD1-F7, were also over-represented in possible loss-of-function variations (Additional file 1: Table S3). Based on these results, one plausible explanation for the phenotypic differences between the two strains and the other strains (low virulence and slow lifecycle) is likely to be the lack of activities of such effectors or digestive proteases, which could lead them to display low ingestion of nutrients and provoke a delay in development. In addition, the effects of unique variations in specific genes could also be important in explaining the different ecological traits of OKD1-F7 and C14-5.

In this study, we could not identify a role for some specific genes found in the low-virulence inbred line S10-P3. However, functional loss of these genes supported the hypothesis that they are associated with the phenotypic differences between S10-P3 and S10-P9. Further research is required to determine the potential role of these genes in pathogenesis. The genes identified and described in this study (Tables 3 and 4, Additional file 1: Table S3, Additional file 1: Table S4 and Additional file 1: Table S8) could be considered as potential factors that explain the mechanism underlying the pathogenesis of PWN.

## Conclusions

By re-sequencing several key strains of *B. xylophilus*, we conducted the first in-depth study of genome-wide variation in *B. xylophilus* populations. Our findings demonstrated that the level of diversity in the *B. xylophilus* genome is high and comparable with that in other hyper-diverse organisms [30] because the geographical range was restricted in this study (only Japanese *B. xylophilus* populations were used). The results presented here highlighted that the level of genomic diversity of PWN was related to its phenotypic variability, including variations in pathogenicity and ecological traits. Moreover, we identified a gene set affected by genomic variation, and functional annotation of those genes indicated that some of them had potential roles in pathogenesis. This study provided an important resource to understand the population structure, pathogenicity and evolutionary ecology of PWN in Japan. This comparative genomics study with geographically diverse *B. xylophilus* populations will greatly accelerate our understanding of the complex evolutionary/epidemic history of this emerging pathogen.

## Methods

### Biological materials and DNA extraction

The origin and ecological features of the nematode isolates or inbred lines used in this study are summarised in Table 1. The *B. xylophilus* genome sequence (ver. 1.2) generated with the Ka4C1 inbred line was used as the genome reference [19]. T4 is an isolate with high virulence. The inbred lines S10-P3 and S10-P9 were derived from the highly virulence isolate S10 [24], but they differ in their virulence to *Pinus* spp. (very low in P3 and high in P9) (Table 1). The F7 inbred lines were derived from a low virulence isolate (OKD-1) [24]. C14-5 is an isolate from the insect *Monochamus alternatus* with low virulence.

### Genomic DNA preparation and sequencing

Nematodes were cultivated for 10 days on *Botrytis cinerea* grown on autoclaved barley grains with antibiotics (100 μg/mL streptomycin and 25 μg/mL chloramphenicol). The nematodes were collected using a modified Baermann funnel technique for 3 h at 25 °C and cleaned by sucrose flotation [32], followed by three rinses with 0.5× PBS. Genomic DNA was extracted from nematodes using QIAamp DNA Mini Kit (Qiagen) for Ka4C1 or GenomeTip-100G (Qiagen) for the others, according to the manufacturer’s instructions. One microgram of the DNA was used to construct standard 300-bp libraries, using a TruSeq DNA Sample Preparation Kit with the standard protocol (Illumina). Libraries were sequenced on an Illumina HiSeq2000, according to the manufacturer’s recommended protocol, to produce 100-bp paired end reads.

### Sequence alignment and SNP calling

Illumina reads for each isolate/line were mapped to the *B. xylophilus* reference genome (ver. 1.2) using SMALT v0.7.4 (https://www.sanger.ac.uk/resources/software/smalt/), with options –x (each mate is mapped independently) and –y 0.8 (minimum 80 % of the read length matches in mapped reads). The mean read depth per base of each sample by Illumina short reads ranges from 14 and 79× (Additional file 1: Table S1). The mapped region of the genome varied from 99.76 % in the reference strain (Ka4C1) to 92.32 % in strain C14-5. Genome positions with a cumulative read coverage of more than 15 across all samples covered 97.44 % of the total genome (72.7 M in 74.6 M bp) and those positions were used for further variant calling.

Variant calling was performed according to the ‘best practice protocol’ in GATK [33]. In brief, reads containing indels were realigned using the Smith-Waterman algorithm (by GATK RealignerTargetCreator and Indel Realigner). Final variant calls were made and false-positives were filtered out using GATK UnifiedGenotyper and VariantFiltration in all samples (including data from the original genome strain). Indels were positioned at the left most position and common bases were trimmed from indels using the GATK LeftAlignAndTrimVariants. From the resulting variant dataset, we generated a stringent dataset for downstream analyses by applying a minim 15× coverage filter, quality by depth minimum of 10 and Fisher strand (Phred-scaled p-value using the Fisher’s Exact Test to detect strand bias) smaller than 20, using the GATK SelectVariants. Depth of coverage was calculated using GATK walker DepthofCoverage.

Variant annotation was performed using [34]. The effects on protein functions of non-synonymous SNPs were predicted using the SIFT algorithm [27] and Align GVGD [28]. Function enrichment analysis was performed in Blast2GO v.2.7.1 [35], using the annotated genome of *B. xylophilus*. Synonymous and non-synonymous polymorphism densities (pS and pN) were calculated for all pairwise comparisons of the six strains. The total number of synonymous and non-synonymous sites in the genome were obtained from *B. xylophilus* gene models (ver. 1.2) and used to calculate the densities.

Large structural changes and CNVs were detected using Cnv-seq (ver. 2014.08.12) [36] with options: −log2-threshold 0.6 − *p*-value 0.001.

Statistical analyses and drawing graphs were performed using the R (www.r-project.org) or an in-house Python script.

### Phylogenetic reconstruction

Both homozygous and heterozygous SNP positions extracted using GATK SelectVariants were used to construct phylogenetic trees. We used the parsimony criterion and tree-bisection reconnection to swap branches in the tree space with PAUP * 4b10 for 100 bootstraps [37], with a hetequal character transition matrix, as previously described by James et al. [38]. Trees were visualised by TreeView [39].

### SNP and indel validation

Validation of SNPs and indels was performed using an aliquot of the DNA solution that was used to generate the Illumina sequencing reads. Primers were designed from flanking regions of each variant using Primer3 v2.3.5 [40]. The PCR primers used for validation are listed in Additional file 1: Table S1. PCR amplifications were performed using GoTaq (Promega) under the following conditions: 3 min at 94 °C; 30 cycles of 30 s at 94 °C, 30 s at 55 °C and 30 s at 72 °C; followed by 5 min at 72 °C. PCR products were purified using a MinElute96 UF PCR Purification Kit (Qiagen) and sequenced in both directions using a BigDye Terminator Sequencing Kit v.3.1 (Applied Biosystems) on a DNA multi-capillary sequencer (Model 3130XL genetic analyser; Applied Biosystems).

### Assembly and annotation of unmapped reads

Reads unmapped for each sample were individually assembled *de novo* using Velvet v.1.2.10 [41] with K-mer 29. To remove any contaminants or artefact, contigs bigger than 200 bp and having more than 50-bp overlapping ends with the reference genome sequences were only kept, as described previously [26]. Augustus v2.5.5 [42] was used to predict protein coding genes in the contigs, using the parameters used in the reference genome construction [19]. Proteins with identities ≥ 90 % were used to create a Venn diagram. Proteins were annotated using Blast2Go v.2.7.1 [35].

## Additional files

Additional file 1:
**Table S1.** Read mapping summary. Reads were mapped to the reference genome (Ka4C1) using Smalt v.0.74 with options –x (more exhaustive search for alignments) and –y = 0.8 (filters output alignments by a threshold in the number of exactly matching nucleotides). **Table S2.** Variant validation using capillary sequencing. *Using reference genome. **Homozygous/heterozygous with the reads mapped. **Table S3.** Fisher enrichment test (*P* < 0.05) performed in Blast2GO v.2.7.0 for Frameshift change variant type and function for all samples. **Table S4.** Fisher enrichment test (*P* < 0.05) performed in Blast2GO v.2.7.0 for Stop change variant type and function for all samples. **Table S5.** Statistics of assemblies of unmapped reads in isolates and inbred lines used in this study. **Table S7.** Structual chnages identified in each strain compared to the reference by cnv-seq. Table S8. Fisher enrichment test (*P* < 0.05) performed in Blast2GO v.2.7.0 for frameshift change and stop change variant type and function for exact variant between C14-5 and OKD1-F7 and different to the other samples. (PDF 173 kb) Additional file 2: Figure S1. Specific variant position for each of the samples studied using the ANNOVAR program. Intergenic: variant is in the intergenic region, not included in Upstream or Downstream, Intronic: variant overlaps an intron, Exonic: variant overlaps a coding region, Upstream: variant overlaps 1-Kb region upstream of the transcription start site, Downstream: variant overlaps 1-Kb region downstream of the transcription end site, Splicing: variant is within 2 bp of a splicing junction. The absolute numbers of variants were shown above the bars. Figure S2. Specific variant effect for each of the samples studied using the ANNOVAR program. Synonymous : a single nucleotide change that does not cause an amino acid change, Non synonymous : a single nucleotide change that causes an amino acid change, Frameshift insertion/deletion: an insertion or deletion of one or more nucleotides that cause frameshift changes to proteins, Stop gain/loss: a nonsynonymous SNP or indel that leads to the immediate creation/elimination of a stop codon at the variant site, Frameshift substitution: a block substitution (not insertion or deletion) of one or more nucleotides that cause frameshift changes, unknown: unknown function (caused by various errors in the gene structure definition in the database file). Each position can have more than one alternative variant and the effect could be different. (PDF 65 kb) Additional file 3: Table S6. Distribution of proteins obtained by *de novo* assembly of unmapped reads in the isolates/inbred lines, including Blast2Go annotations. 1: presence/0: absence. (XLSX 18 kb)
