# Supplementary material for: Genome-wide variation in the pinewood nematode Bursaphelenchus xylophilus and its relationship with pathogenic traits
Source: BMC Genomics. 2015 Oct 23;16:845. doi: 10.1186/s12864-015-2085-0 (PMC4619224; doi:10.1186/s12864-015-2085-0)
Supplement: Additional file 1: — Table S1. Read mapping summary. Reads were mapped to the reference genome (Ka4C1) using Smalt v.0.74 with options –x (more exhaustive search for alignments) and –y = 0.8 (filters output alignments by a threshold in the number of exactly matching nucleotides). Table S2. Variant validation using capillary sequencing. *Using reference genome. **Homozygous/heterozygous with the reads mapped. Table S3. Fisher enrichment test (P < 0.05) performed in Blast2GO v.2.7.0 for Frameshift change variant type and function for all samples. Table S4. Fisher enrichment test (P < 0.05) performed in Blast2GO v.2.7.0 for Stop change variant type and function for all samples. Table S5. Statistics of assemblies of unmapped reads in isolates and inbred lines used in this study. Table S7. Structual chnages identified in each strain compared to the reference by cnv-seq. Table S8. Fisher enrichment test (P < 0.05) performed in Blast2GO v.2.7.0 for frameshift change and stop change variant type and function for exact variant between C14-5 and OKD1-F7 and different to the other samples. (PDF 173 kb) [file 12864_2015_2085_MOESM1_ESM.pdf]

Table S1. Read mapping summary. Reads were mapped to the reference genome using smalt v.0.74 with –x (more exhaustive search for alignments) and –y = 0.8 (filters output alignments by a threshold of the number of exactly matching nucleotides).

Strains	Mapped genome region (%)	Number of mapped reads	Mean coverage	Median coverage	% bases > coverage 15
**C14-5**	92.32	10682103	14.54	14	40.0
**OKD-1-F7**	93.26	18686713	25.47	23	78.3
**S10-P3**	95.64	18716664	25.62	25	83.3
**S10-P9**	95.61	18299752	25.07	25	84.7
**T4**	95.68	14533880	19.91	19	72.9
**Ka4C1**	99.76	57312623	78.99	74	98.7

Table S2. Variant validation by PCR and Sanger sequencing. *position in reference genome.

Variant position*	Strains	Primers	Variant type	Correct
scaffold00036:318731	Ka4C1, C14-5, OKD1-F7	F: 5’ TACAAGCGTGACGTTGAGGTG3’<br>R:5’ GTGCTGGCGAATGGGTATCC3’	SNP	Yes
scaffold00055:149186	Ka4C1, C14-5, OKD1-F7	F: 5’ GGTGATAGACCATCTGAAG3’<br>R: 5’ CATCATGATGTCTCTTCATAT3’	SNP	Yes
scaffold00055:149187	Ka4C1, C14-5, OKD1-F7	F: 5’ GGTGATAGACCATCTGAAG3’<br>R: 5’ CATCATGATGTCTCTTCATAT3’	SNP	Yes
scaffold00351:1024128	Ka4C1, C14-5, OKD1-F7	F: 5’TCTTATAGTGTGCCTAGTAC3’<br>R: 5’GCCTGCAGGTCCTCAACTTCA3’	SNP	Yes
scaffold00713:1509715	Ka4C1, C14-5, OKD1-F7	F: 5’TGGCTGATCCTATTATTTACAG3’<br>R: 5’ACCTCAGATCAACGGTTATCG3’	SNP	Yes
scaffold01144:774070	Ka4C1, C14-5, OKD1-F7	F: 5’AGTTATCGATATTAACATTCCTG3’<br>R: 5’AATCTCCATTGGTAGACGTCC3’	SNP	Yes
scaffold01144:774074	Ka4C1, C14-5, OKD1-F7	F: 5’AGTTATCGATATTAACATTCCTG3’<br>R: 5’AATCTCCATTGGTAGACGTCC3’	SNP	Yes
scaffold01149:107366	Ka4C1, C14-5, OKD1-F7	F: 5’AGGAGAATGAACCACACTTTCG3’<br>R: 5’AGTGTGGAATTCGAATCATCGC3’	SNP	Yes
scaffold01149:107438	Ka4C1, C14-5, OKD1-F7	F: 5’AGGAGAATGAACCACACTTTCG3’<br>R: 5’AGTGTGGAATTCGAATCATCGC3’	SNP	Yes
scaffold00116:953732	Ka4C1, S10-P3, S10-P9	F: 5’CCGTGGTTACTACTCGGTTGA3’<br>R: 5’CGAGATTCGTGGTTACCTCTG3’	SNP	Yes
scaffold00116:954049	Ka4C1, S10-P3, S10-P9	F: 5’CCGTGGTTACTACTCGGTTGA3’<br>R: 5’CGAGATTCGTGGTTACCTCTG3’	SNP	Yes
scaffold00119345928	Ka4C1, S10-P3, S10-P9	F: 5’GGCGCTTCTACAAGATCCAGCT3’<br>R: 5’GCCTTCACATCCGTCACC3’	SNP	Yes
scaffold00333:638952	Ka4C1, S10-P3, S10-P9	F: 5’CCTCATTGCTAAGATCCTCCA3’<br>R: 5’AGCAACACTCGGGGAGAAG3’	SNP	Yes
scaffold00333:641298	Ka4C1, S10-P3, S10-P9	F: 5’GCTACCGTAACCCAAAAGGA3’<br>R: 5’TTGGACTTGCTGAAGTAGGTGA3’	SNP	Yes
scaffold00422:1624112	Ka4C1, S10-P3, S10-P9	F: 5’GGAGTCGTTCGTCCAATGTT3’<br>R: 5’ACCTCGCGCATTGGTTAAT3’	SNP	Yes

scaffold00460:179854	Ka4C1, S10-P3, S10-P9	F: 5’AGCGCTGTGAAGGAGGAGAG3’<br>R: 5’CCAAGCATTACAGCAATACACC3’	SNP	Yes
scaffold00713:1183294	Ka4C1, S10-P3, S10-P9	F: 5’ CGCTCTTGTTGATTGTGTGG3’<br>R: 5’TCCTCATTCTCGGCTCTGAT3’	SNP	Yes
scaffold00770:367968	Ka4C1, S10-P3, S10-P9	F: 5’CTTGCACGGGGGTATATTTG3’<br>R: 5’TGCCGCTTAACAGATCCTTC3’	SNP	Yes
scaffold01139:25976	Ka4C1, S10-P3, S10-P9	F: 5’ CAGAGATCGTCTTTGCACCA3’<br>R: 5’CCGCACTGGTTCTCCATTAT3’	SNP	Yes
scaffold01198:804265	Ka4C1, S10-P3, S10-P9	F: 5’ ATCGTGGGCTGTCTTGAAAC3’<br>R: 5’TTCGAATAGCGGTGGTCAAT3’	SNP	Yes

scaffold00116:1209822	Ka4C1, S10-P3, S10-P9	F: 5’ATTCAGCTGCAAATGGGAAC3’<br>R:5’TGCTTGAATCTTTCCCTCCA3’	INDEL	Yes
scaffold01133:2211	Ka4C1, S10-P3, S10-P9	F: 5’ TCACCGACCAAAGTTCATTC3’<br>R:5’ ACAATCGATGCCTTCTTGCT3’	INDEL	Yes
scaffold01144:1209693	Ka4C1, S10-P3, S10-P9	F: 5’ GCCTCAATGGGTTAAAGTGG3’<br>R: 5’ TGAGCCAAGCCTTTCTTGAT3’	INDEL	Yes
contig01495:1422	Ka4C1, C14-5, OKD1-F7	F: 5’ACAATTTTACCATRCTTTGATC3’<br>R:5’TGCCACTGGATCAGTCCCGCAGT3’	INDEL	Yes
scaffold00036:318754	Ka4C1, C14-5, OKD1-F7	F: 5’ TACAAGCGTGACGTTGAGGTG3’<br>R:5’ GTGCTGGCGAATGGGTATCC3’	INDEL	Yes
scaffold00055:149167	Ka4C1, C14-5, OKD1-F7	F: 5’ GGTGATAGACCATCTGAAG3’<br>R: 5’ CATCATGATGTCTCTTCATAT3’	INDEL	Yes
scaffold00351:1024128	Ka4C1, C14-5, OKD1-F7	F: 5’TCTTATAGTGTGCCTAGTAC3’<br>R: 5’GCCTGCAGGTCCTCAACTTCA3’	INDEL	Yes
scaffold00351:1557328	Ka4C1, C14-5, OKD1-F7	F: 5’ACAGCCTTCTTGGCGAGCCTCTG3’<br>R: 5’CATAGCACATAACCATCCTAT3’	INDEL	Homo/Hetero
scaffold00364:1335971	Ka4C1, C14-5, OKD1-F7	F: 5’CGGTGAGCTCCACACTTATCC3’<br>R: 5’AGAGCCGGAGTTGCAGCTATC3’	INDEL	Yes
scaffold00713:1509727	Ka4C1, C14-5, OKD1-F7	F: 5’TGGCTGATCCTATTATTTACAG3’<br>R: 5’ACCTCAGATCAACGGTTATCG3’	INDEL	Yes
scaffold00713:2666839	Ka4C1, C14-5, OKD1-F7	F: 5’TTCTACAACATCATTGTCTCGC3’<br>R: 5’ATGACTGTGAAGCTGAAGGCTC3’	INDEL	Yes
scaffold00713:2774985	Ka4C1, C14-5, OKD1-F7	F: 5’TCATTGCACTTTATGGATGAC3’	INDEL	Yes

		R: 5'ACATCAGTGTTAATGGCACTAC3'		
scaffold01144:774059	Ka4C1, C14-5, OKD1-F7	F: 5'AGTTATCGATATTAACATTCCTG3'<br>R: 5'AATCTCCATTGGTAGACGTCC3'	INDEL	Yes
scaffold01149:107377	Ka4C1, C14-5, OKD1-F7	F: 5'AGGAGAATGAACCACACTTTCG3'<br>R: 5'AGTGTGGAATTCGAATCATCGC3'	INDEL	Yes

Table S3. Enriched GO terms affected by frameshift change variants in each strain

GO-ID	Term	P-value
**OKD1-F7**		
GO:0004222	Metalloendopeptidase activity	6.03E-04
GO:0004190	Aspartic-type endopeptidase activity	0.00128878
GO:0015440	Peptide-transporting ATPase activity	0.00179376
GO:0004869	Cysteine-type endopeptidase inhibitor activity	0.006069
GO:0016810	Hydrolase activity, acting on carbon-nitrogen (but not peptide) bonds	0.00804591
**C14-5**		
GO:0004222	Metalloendopeptidase activity	1.36E-04
GO:0004190	Aspartic-type endopeptidase activity	0.00105512
GO:0015440	Peptide-transporting ATPase activity	0.00168354
GO:0005247	Voltage-gated chloride channel activity	0.00435883
GO:0004123	Cystathionine gamma-lyase activity	0.004913
GO:0004869	Cysteine-type endopeptidase inhibitor activity	0.00542517
**T4**		
GO:0008237	Metallopeptidase activity	0.0019977
GO:0015086	Cadmium ion transmembrane transporter activity	0.00518056
**S10-P3**		
GO:0005247	Voltage-gated chloride channel activity	0.00661501
GO:0004869	Cysteine-type endopeptidase inhibitor activity	0.0078037
**S10-P9**		
GO:0005247	Voltage-gated chloride channel activity	0.00661501
GO:0004869	Cysteine-type endopeptidase inhibitor activity	0.0078037

Table S4. Enriched GO terms (p<0.05) affected by Stop codon change variants in each sample

GO-ID	Term	P-value
**OKD1-F7**		
GO:0004555	Alpha, alpha-trehalase activity	0.00169259
GO:0008237	Metallopeptidase activity	0.00531556
GO:0004643	Phosphoribosylaminoimidazolecarboxamide formyltransferase activity	0.00960912
GO:0003937	IMP cyclohydrolase activity	0.00701652
**C14-5**		0.00531556
GO:0004555	Alpha, alpha-trehalase activity	0.00183127
GO:0008237	Metallopeptidase activity	0.00629035
GO:0015248	Sterol transporter activity	0.00747817
GO:0005509	Calcium ion binding	0.0096447
**T4**		
GO:0010181	FMN binding	0.000491269
**S10-P3**		
GO:0010181	FMN binding	0.00650591
GO:0004889	Acetylcoline-activated cation-selective channel activity	0.00748279
GO:0004500	Dopamine beta-monooxygenase activity	0.00979577
**S10-P9**		
GO:0010181	FMN binding	0.00650591
GO:0004889	Acetylcoline-activated cation-selective channel activity	0.00748279
GO:0004500	Dopamine beta-monooxygenase activity	0.00979577

Table S5. Statistics of assemblies of unmapped reads in the isolates and inbred lines used in this study.

	Strains				
	**C14-5**	**OKD-1-F7**	**S10-P3**	**S10-P9**	**T4**
**Total assemble length**	342305	337871	310717	329596	260975
**Number of contigs**	648	601	599	623	491
**Mean contig length**	528.25	562.18	518.73	529.05	531.51
**Longest contig**	16528	25594	38899	58705	19735
**Contig N50**	608	605	536	533	565
**Contig N50n**	123	86	105	95	81
**Number of predicted genes**	40	29	25	33	30

Table S7. Big structual chnages identified in each strain compared to the reference by cnv-seq.

	C145	OKD1-F7	S10-P3	S10-P9	T4
CNV percentage in genome (%)	4.0	6.4	5.5	4.2	2.9
CNV nucleotide content (b)	3053860	4811840	4140592	3147530	2207600
CNV count	978	2347	2128	1519	881
Mean size (b)	3123	2050	1946	2072	2506
Median size (b)	8215	7520	6399	7498	7200
Max Size (b)	25440	31680	16319	26406	16400
Min Size (b)	2120	1280	1279	1304	1600

Table S8. Enriched GO terms ($P < 0.05$) affected by frameshift change and stop codon change variants common in C14-5/OKD1-F7 and different in the other samples.

**FRAMESHIFT CHANGE**		
**GO-ID**	**Term**	**P-value**
GO:0004222	metalloendopeptidase activity	4.08E-4
GO:0015440	peptide-transporting ATPase activity	4.69E-4
**STOP CHANGE**		
**GO-ID**	**Term**	**P-value**
GO:0008307	structural constituent of muscle	0.0043
GO:0004175	endopeptidase activity	0.0047
