# Supplementary figures and images for: Genome-wide variation in the pinewood nematode Bursaphelenchus xylophilus and its relationship with pathogenic traits
Source: BMC Genomics. 2015 Oct 23;16:845. doi: 10.1186/s12864-015-2085-0 (PMC4619224; doi:10.1186/s12864-015-2085-0)

Figure S1

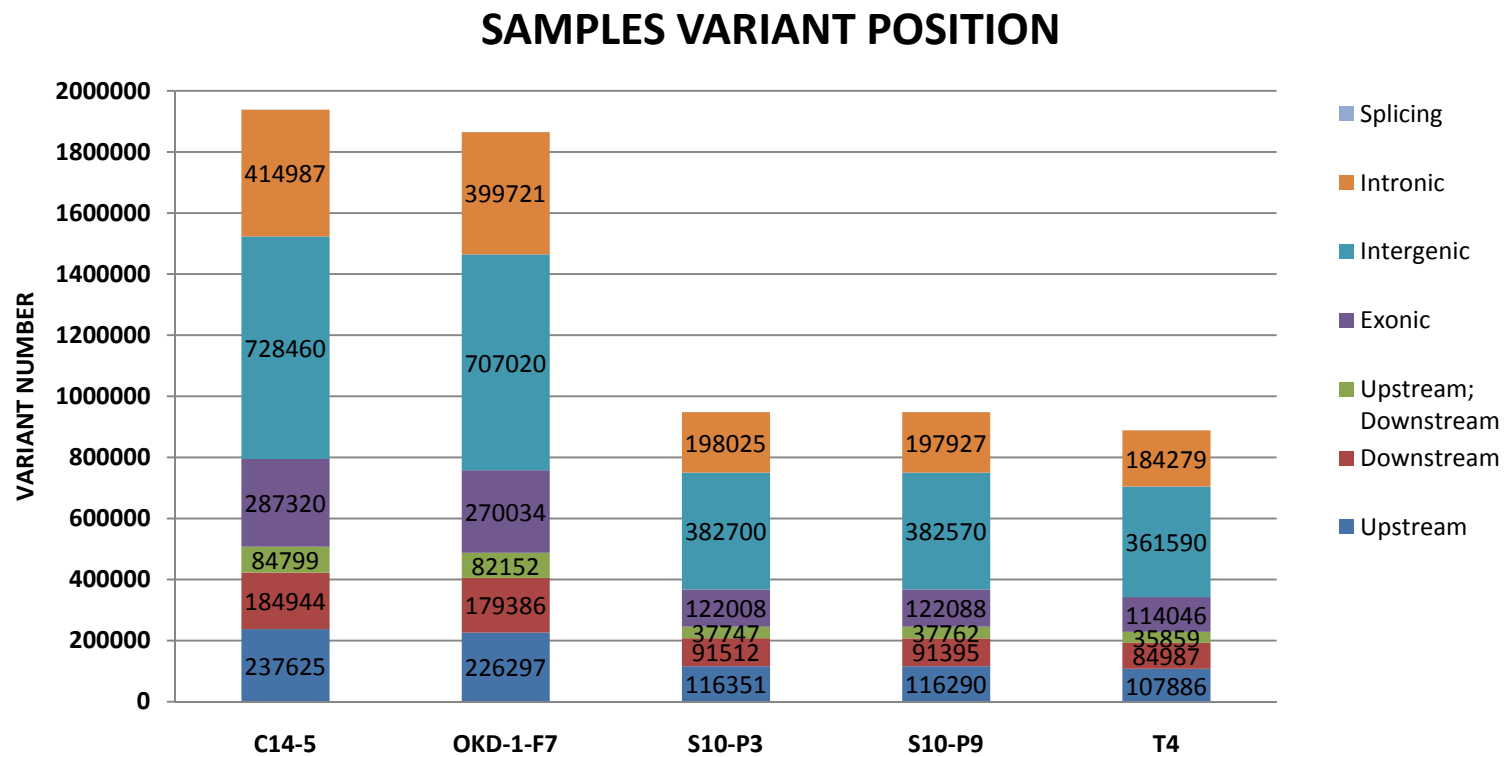

Figure S2

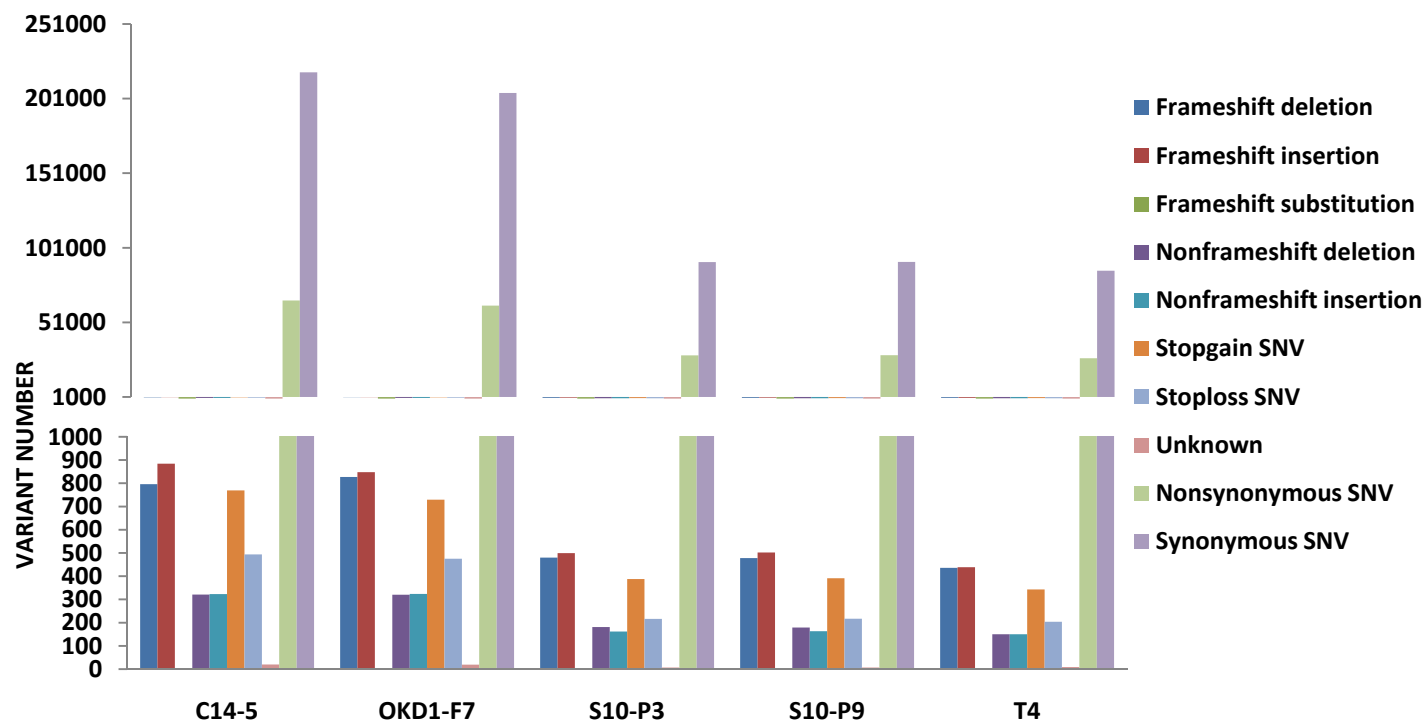

Supplement: Additional file 2: Figure S1. — Specific variant position for each of the samples studied using the ANNOVAR program. Intergenic: variant is in the intergenic region, not included in Upstream or Downstream, Intronic: variant overlaps an intron, Exonic: variant overlaps a coding region, Upstream: variant overlaps 1-Kb region upstream of the transcription start site, Downstream: variant overlaps 1-Kb region downstream of the transcription end site, Splicing: variant is within 2 bp of a splicing junction. The absolute numbers of variants were shown above the bars. Figure S2. Specific variant effect for each of the samples studied using the ANNOVAR program. Synonymous : a single nucleotide change that does not cause an amino acid change, Non synonymous : a single nucleotide change that causes an amino acid change, Frameshift insertion/deletion: an insertion or deletion of one or more nucleotides that cause frameshift changes to proteins, Stop gain/loss: a nonsynonymous SNP or indel that leads to the immediate creation/elimination of a stop codon at the variant site, Frameshift substitution: a block substitution (not insertion or deletion) of one or more nucleotides that cause frameshift changes, unknown: unknown function (caused by various errors in the gene structure definition in the database file). Each position can have more than one alternative variant and the effect could be different. (PDF 65 kb) [file 12864_2015_2085_MOESM2_ESM.pdf]
